# Comparative evaluation of modern dosimetry techniques near low‐ and high‐density heterogeneities

**DOI:** 10.1120/jacmp.v16i5.5589

**Published:** 2015-09-08

**Authors:** Eyad A. Alhakeem, Sami AlShaikh, Anatoly B. Rosenfeld, Sergei F. Zavgorodni

**Affiliations:** ^1^ Department of Physics and Astronomy University of Victoria Victoria BC Canada; ^2^ Department of Medical Physics British Columbia Cancer Agency–Vancouver Island Centre Victoria BC Canada; ^3^ Centre for Medical Radiation Physics University of Wollongong Wollongong New South Wales 2522 Australia; ^4^ Ministry of Health Riyadh Kingdom of Saudi Arabia; ^5^ Ministry of Education Riyadh Kingdom of Saudi Arabia

**Keywords:** Monte Carlo dose calculation, Acuros AXB, Gafchromic EBT2 film, MOS*kin*, interface dosimetry

## Abstract

The purpose of this study is to compare performance of several dosimetric methods in heterogeneous phantoms irradiated by 6 and 18 MV beams. Monte Carlo (MC) calculations were used, along with two versions of Acuros XB, anisotropic analytical algorithm (AAA), EBT2 film, and MOS*kin* dosimeters. Percent depth doses (PDD) were calculated and measured in three heterogeneous phantoms. The first two phantoms were a $30\times 30\times 30\;{\text{cm}}^{3}$ solid‐water slab that had an air‐gap of $20\times 2.5\times 2.35\;{\text{cm}}^{3}$. The third phantom consisted of $30\times 30\times 5\;{\text{cm}}^{3}$ solid water slabs, two $30\times 30\times 5\;{\text{cm}}^{3}$ slabs of lung, and one $30\times 30\times 1\;{\text{cm}}^{3}$ solid water slab. Acuros XB, AAA, and MC calculations were within 1% in the regions with particle equilibrium. At media interfaces and buildup regions, differences between Acuros XB and MC were in the range of +4.4% to −12.8%. MOS*kin* and EBT2 measurements agreed to MC calculations within ∼2.5%, except for the first centimeter of buildup where differences of 4.5% were observed. AAA did not predict the backscatter dose from the high‐density heterogeneity. For the third, multilayer lung phantom, 6 MV beam PDDs calculated by all TPS algorithms were within 2% of MC. 18 MV PDDs calculated by two versions of Acuros XB and AAA differed from MC by up to 2.8%, 3.2%, and 6.8%, respectively. MOS*kin* and EBT2 each differed from MC by up to 2.9% and 2.5% for the 6 MV, and by −3.1% and ∼2% for the 18 MV beams. All dosimetric techniques, except AAA, agreed within 3% in the regions with particle equilibrium. Differences between the dosimetric techniques were larger for the 18 MV than the 6 MV beam. MOS*kin* and EBT2 measurements were in a better agreement with MC than Acuros XB calculations at the interfaces, and they were in a better agreement to each other than to MC. The latter is due to their thinner detection layers compared to MC voxel sizes.

PACS numbers: 87.55.K‐, 87.55.kd, 87.55.km, 87.53.Bn, 87.55.k

## I. INTRODUCTION

Modern radiotherapy treatment techniques are developing rapidly and continuously, opening the doors for more complex patient treatments. Such complexity adds more challenges on treatment planning (TP) dose calculation algorithms. Historically, dose calculation algorithms improved significantly from simple dose correction‐based methods to advanced convolution/superposition calculations and, further, to linear Boltzmann transport equation (LBTE) solutions. Developments in TP algorithms are always limited by the necessity of getting the calculations within an acceptable time window, which may compromise the calculation accuracy.

Monte Carlo (MC) method calculates the dose using random sampling of the particle state during its transport in a medium. It has been accepted as a “gold standard” in dose calculations^1^, ^2^ and arguably is comparable to experimental measurements in terms of reliability of its dose estimates.

Convolution/superposition is probably the most commonly used group of algorithms in modern TP dose calculations. Their implementations, such as anisotropic analytical algorithm (AAA),^3^, ^4^, ^5^ where the lateral electron/photon scatter component is modeled as a variable in different directions, a considerably improved calculation accuracy compared to previously used pencil beam convolution algorithms.^6^, ^7^ However, AAA is still not able to accurately calculate doses at extreme density interfaces. Aarup et al.^(8)^ reported that discrepancies between AAA and BEAMnrc/DOSXYZnrc dose calculations increased as lung density decreased from 0.4 $g/{\text{cm}}^{3}$ to 0.01 $g/{\text{cm}}^{3}$. The differences for the lowest clinically meaningful lung density of 0.1 $g/{\text{cm}}^{3}$ were up to 5.9% and 8.9% when using 6 and 18 MV beams, respectively. The differences exceeded 10% and 30% for 0.01 $g/{\text{cm}}^{3}$ lung density for the 6 and 18 MV energies, respectively. Chow et al.^(9)^ evaluated AAA and collapsed cone convolution (CCC) algorithm against MC for oblique tangential photon beams and showed, in some cases, differences of up to 18.0%±1.3% and 8.3%±1.8% for AAA and CCC, respectively.

The Acuros XB dose calculation algorithm, released by Varian Medical System for the Eclipse treatment planning system (Varian Medical Systems, Palo Alto, CA), is the grid‐based LBTE solver that models particles fluence transport in a medium. It provides a deterministic solution for the Boltzmann equation, unlike the MC approach, where the solution is achieved stochastically. Acuros XB was shown to be more accurate than AAA and CCC in calculating the dose in regions with complex geometries and heterogeneities.^10^, ^11^, ^12^, ^13^, ^14^, ^15^, ^16^, ^17^, ^18^, ^19^, ^20^ Bush et al.^(10)^ validated Acuros XB against MC in multi‐slab heterogeneous phantoms with low‐ and high‐density heterogeneities. Calculated PDD and lateral profiles demonstrated superiority of Acuros XB over AAA. In this study, a maximum discrepancy of 4.5% compared to MC was observed near the air cavity interface. However, for most calculations, Acuros XB was within 2.9% of MC compared to 17.5% difference when using AAA.

A number of experimental dose measurements have been conducted in heterogeneous structures to validate modern TP dose algorithms.^10^, ^12^, ^13^, ^15^, ^16^, ^17^, ^18^, ^19^, ^20^, ^21^ The size of detectors and, especially, the thickness of its sensitive layer must be as small as possible, due to the steep dose gradient at the media interface. Gafchromic films,^22^, ^23^ Metal Oxide Silicon Field Effect Transistor (MOSFET) and, partially, thermoluminescent dosimeters (TLD) detectors satisfy these criteria. Hoffmann et al.^(14)^ used Gafchromic films (EBT) in heterogeneous media (CIRS IMRT Thorax Phantom) and compared the measured doses with Acuros XB and AAA calculations. In that study, 22 different treatment plans were measured and calculated. The mean values of the percentage passing rate (3% / 3 mm criteria) were found to be 98.2%±1.1% and 99.5%±0.3% for Acuros XB using 6 and 15 MV energy beams, respectively, while a passing rate of 94.1%±7.0% and 96.1%±3.3% for the 6 and 15 MV energies were observed for AAA, respectively. Kan et al.^(13)^ investigated the accuracy of Acuros XB near air/tissue interfaces using small fields ($2\times 2.5\times 5\;{\text{cm}}^{2}$). PDDs calculated for the $2\times 2\;{\text{cm}}^{2}$ field were overestimated when compared to TLD measurements at the air/tissue interface by 41% and 6% for AAA and Acuros XB, respectively. In another paper, Kan et al.^(19)^ used Gafchromic EBT3 films and TLD to compare Acuros XB and AAA calculations against EBT3 and TLD measurements adjacent to air and bone inserts in a rectangular tissue phantom. The average dose difference (calculated data ‐ measured data) for all the tested cases in this study were 4.3%. Another study by Carrasco et al.^(24)^ involved comparing five TP dose calculation algorithms against MC simulation, MOSFET and TLD measurements in multilayer slab phantom with cortical bone used as high‐density heterogeneity. In that study, TLD measurements underestimated MC calculations by 5.7%±1.1% near the exit interface. Ding et al.^(25)^ found that AAA calculations near water–lung interfaces agree with MC calculation and MOSFET measurements for 6 and 18 MV photon beams within experimental and statistical uncertainties (1%–3%).

Kwan et al.^(26)^ validated a special design of MOSFET detector, known as MOS*kin* ^(26)^ (CMRP, Wollongong, Australia), for surface measurements and found them to be within 2% compared to the Attix parallel plate ionization chamber. Qi et al.^(27)^ used MOS*kin* to evaluate commercial TPS (Corvus 6.2) in calculating superficial dose and found that calculated dose overestimated MOS*kin* measurements by an average of 7.8%.

In this study, Gafchromic EBT2 film (Ashland, Specialty Ingredients, Wayne, NJ) and MOS*kin* detectors, as well as MC calculations, were used to estimate the dose near extreme media heterogeneities irradiated by 6 and 18 MV beams of different sizes. Water–air, water–steel, and water–lung interfaces were used, and the measured dose was compared to MC calculations, as well as to AAA and Acuros XB predictions. This combination of experimental and MC methods allowed testing accuracy of commercial algorithms and it also allowed evaluation of accuracy and consistency of “benchmarks” —experimental measurements and MC in extreme conditions.

## II. MATERIALS AND METHODS

### A. Experimental setup

**Figure 1:**
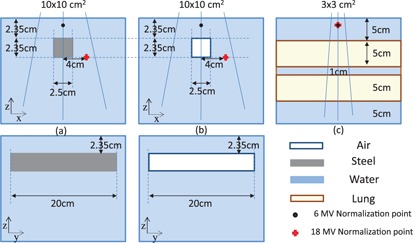
Diagrams of the three phantoms created to measure dose profiles: (a) shows geometry of the phantom with water‐steel‐water interface; (b) shows geometry of the phantom with water‐air‐water interface; and (c) shows geometry of the phantom with water‐lung‐water interface. Field sizes used for irradiating each phantom are also shown. Varian 21EX 6 and 18 MV were used in these measurements and calculations. Measured and calculated percent depth doses were normalized at the points shown in the diagram.

Three different phantoms with high/low density heterogeneities, as shown in Fig. 1, were made (virtually and experimentally) to compare the performance of the five dosimetric techniques used in this study. The first phantom was a $30\times 30\times 30\;{\text{cm}}^{3}$ solid water slab that had an air cavity of $20\times 2.5\times 2.35\;{\text{cm}}^{3}$. This was created to imitate the water–air heterogeneity encountered in clinical situations, such as head and neck treatments. The second phantom had exactly the same geometry with a steel rod ($\rho =7.8\;g/{\text{cm}}^{3}$) of $20\times 2.5\times 2.35\;{\text{cm}}^{3}$ size inserted to fill the air cavity. This phantom was used to measure the dose near a high‐density heterogeneity and evaluate performance of the dose calculations. The third phantom was made with two $30\times 30\times 5\;{\text{cm}}^{3}$ slabs of solid water, two $30\times 30\times 5\;{\text{cm}}^{3}$ slabs of lung, and one $30\times 30\times 1\;{\text{cm}}^{3}$ slab of solid water stack, as shown Fig. 1(c). This phantom was designed to simulate a lung tumor. For simplicity, those phantoms will be referred as water–air, water–steel, and water– lung phantoms, respectively. Phantom slabs used in this study were Gammex (Gammex RMI, Middleton, WI) “Solid Water” RMI‐457 (mass density $\rho =1.046\;g/{\text{cm}}^{3}$) and “Lung” LN300 RMI‐455 ($\rho =0.3\;g/{\text{cm}}^{3}$).

**Figure 2:**
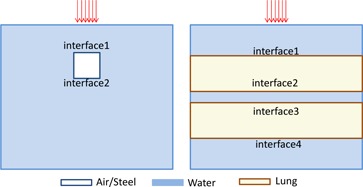
Diagram labeling the interfaces between the different mediums in (left) water–steel/air and (right) water–lung phantoms.

A Varian 21EX (Varian Oncology Systems, Palo Alto, CA) linac was used to expose the phantoms to 6 and 18 MV photons. The water–air and water–steel phantoms were irradiated by $10\times 10\;{\text{cm}}^{2}$ field beams at 100 cm SSD and the lung–water phantom was irradiated by a $3\times 3\;{\text{cm}}^{2}$ field at 89.5 cm SSD with the beam isocenter located at the center of a 1 cm water slab. Percent depth‐dose (PDD) measurements were taken along the beam central axis using EBT2 films and the MOS*kin* detector with computerized reader was used to measure the dose at the water side of the interfaces. This will be described in detail in the following sections. The PDD was normalized at depth of maximum dose ($d_{\text{max}}$) for the 6 MV beam and at an off‐axis point located 4 cm off the beam central axis and depth of 4.7 cm for the 18 MV beam. This point was chosen in a uniform dose region to avoid charged particle disequilibrium conditions. Normalization points are shown in Fig. 1. Figure 2 shows labeling of the interfaces between different media in the three phantoms.

### B. Monte Carlo calculations

Monte Carlo simulations were performed using the Vancouver Island Monte Carlo (VIMC)^28^, ^29^, ^30^ system. VIMC is a Web‐based platform that facilitates the use of BEAMnrc/DOSXYZnrc^31^, ^32^ MC models to simulate transport of photon or electron beams through a patient or phantom geometry. The 6 and 18 MV photon beams from Varian 21EX Clinac were used in this study. The accelerators were modeled according to the manufacturer specifications of the geometries. Photon (PCUT) and electron (ECUT) cutoff energies of 0.01 MeV and 0.700 MeV, respectively, were selected for all calculations. Pretarget electron source with monoenergetic electron energy of 6.0 MeV and 18.5 MeV were configured for the 6 MV and the 18 MV models, respectively. Incident electrons were defined as a symmetric Gaussian intensity distribution with full width half maximum (FWHM) of 0.75 and 1.3 mm energy for the 6 and 18 MeV, respectively. The BEAMnrc models for 6 and 18 MV 21EX beams used in this work have been previously established and benchmarked.^6^, ^10^, ^33^, ^34^, ^35^ Statistical uncertainties for all calculations were less than 1% in all but the air‐filled regions.

Customized phantoms were built through VIMC graphical user interface that is similar to DOSXYZnrc GUI. This interface allows building phantoms with exact geometrical boundaries, avoiding voxelization artifacts commonly present in CT‐based phantoms. The material densities for steel, air, water, and lung were matched to those used for Acuros XB in the Eclipse TPS. Central axis PDDs, as well as lateral profiles, were scored in 0.1 cm voxels. PDD scoring resolution was increased to 0.05 cm for the first three voxels directly above and immediately below each interface of the modeled phantoms.

### C. Acuros XB and AAA calculations

Acuros XB and AAA share the same multiple‐source photon beam source model. It consists of primary photon source, extrafocal photon source, electron contamination source, and photons scattered from wedges. Even though Acuros XB and AAA share the same multiple‐source model, the model parameters are different due to the differences in the dose calculation.^11^, ^36^

Open‐field beam data, required in the configuration process, were acquired using an IC‐15 (IBA Dosimetry, Bartlett, TN) ionization chamber in a Wellhofer (IBA Dosimetry) 48.$0\times 48.0\times 48.0\;{\text{cm}}^{3}$ water tank. These measurements were taken during a departmental commissioning process for 21EX Varian linear accelerator.^(10)^

In this study, AAA version 10.0.28 was used. Acuros XB calculations were performed using two versions of this software, 11.0.02 and 11.0.31, which will be referred to below as AXB1102 and AXB1131, respectively. New Acuros XB version had several updates.^(37)^ Amongst them were: reduced electron cutoff energies (from 500 KeV to 200 KeV); improved photon ray tracing and electron contaminant source; “transport correction” implemented to improve accuracy; resampling to the calculation grid was improved for the voxels that cross structure boundaries.

Three multislab heterogeneous phantoms described in previous sections and shown in Fig. 1, have been created within Eclipse planning software, using contouring tools. The phantoms were created with the exact dimensions of the real phantoms. Material densities, matching those used in DOSXYZnrc, were assigned to the phantom structures manually. Densities of 0.0012 $g/{\text{cm}}^{3}$, 7.8 $g/{\text{cm}}^{3}$, and 0.3 $g/{\text{cm}}^{3}$ were assigned to air, steel, and lung structures, respectively. The calculations were scored in a 0.1 cm grid voxel size with the heterogeneity correction option turned on for all used algorithms (AAA, AXB1102, and AXB1131). PDDs were extracted throughout the beam central axis (CAX) using Eclipse tools. Lateral dose profiles were extracted from water–air and steel–water phantoms. These profiles run through the CAX in x‐axis direction at the depth of 3.5 cm (through water–air and steel–water heterogeneities). AXB calculations, both versions, were reported in the dose to medium ($D_{m}$) mode. AAA calculations were performed and reported in the dose to water ($D_{w}$) mode — the only option available for this algorithm in Eclipse TPS. Dose within steel has not been discussed in this work, as it is irrelevant in clinical practice and AAA was not designed to calculate the dose in steel (unlike MC and Acuros XB).

### D. Gafchromic EBT2 film measurements

Gafchromic EBT2 film was used in this paper. EBT2 has a wide range of dose linearity (1 cGy–40 Gy) and are near‐tissue equivalents. This is an advantage when measuring doses at high‐gradient regions, such as boundaries of heterogeneities, and small fields where detector perturbation is a problem.

**Figure 3:**
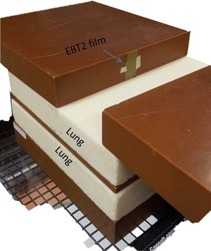
A photograph of the phantom used to measure PDD using Gafchromic EBT2 strips. A piece of film attached to the solid water slab along the beam central axis is shown.

The film dosimetry protocol implemented in this study was based on the manufacturer's recommendations and previous publications on EBT and EBT2 films.^22^, ^23^, ^38^, ^39^, ^40^, ^41^, ^42^, ^43^, ^44^, ^45^, ^46^, ^47^, ^48^, ^49^, ^50^ Film strips of 2 cm width were used to score the depth doses before and after the interfaces, by being placed vertically (Fig. 3) along the beam axis. This way, PDD through the media interface is scored using either one piece of film strip in the case of the water‐air phantom or two pieces of strips in the case of the water‐steel phantom. In the water–lung phantom, four pieces were used: one strip before and after each interface. Effect of air gap on each side of the film strip is ignored in our measurements, and this was validated by comparing PDD measured using 2 cm strip of EBT2 stack between two water slabs against Eclipse (AAA) calculations. The differences between EBT2 and Eclipse beyond the $d_{\text{max}}$ were within ±1%.

The dose measured within the air slab has been converted to dose‐to‐medium by applying stopping power ratio factor, as proposed by Siebers et al.^(51)^ For the 18 MV beam, another piece of film was used at 4.0 cm of the central beam axis and at 4.7 cm depth, as illustrated in Fig. 1.

#### D.1 Film calibration

A sheet of film has been cut into 13 pieces each $5\times 5\;{\text{cm}}^{2}$. Films were then exposed to known doses ranging from 0–6 Gy. To minimize film nonuniformity, the films were scanned before and after exposure and the net optical density (netOD) was calculated by subtracting backgrounds on a piece‐by‐piece basis. After at least 24 hrs, the films were scanned and then the net optical densities were calculated, as described in the following sections. A calibration curve between delivered dose (D) and measured netOD was generated using the analytical form $D_{fit}=a.netOD+b.netOD_{n}$, as outlined in Devic et al.^(47)^

#### D.2 Film scanning

An Epson 10000XL (Epson America, Inc., Long Beach, CA) flatbed document scanner was used to scan the films as per manufacturer scanning protocol and recommendations. The scanner was allowed a ∼15 min warm‐up by doing “preview scans”. All films used for measurements were scanned three times before and after exposure in order to minimize scanning noise.^45^, ^47^ Also, to minimize scanner lateral positioning dependency, a plastic mask was used to reproduce film positioning after the exposure and to prevent them from touching the scanner glass surface and thereby avoiding Newtonian's rings.^(45)^ Films were scanned at least 24 hrs after exposure. Epson software was used for scanning the films in a transmission mode with a resolution of 75 dpi and all image enhancements being turned off. The images were saved as TIFF with 48 bits for further analysis.

#### D.3 Image processing

Film images processed using an in‐house MATLAB (MathWorks, Natick, MA) code that filters (using a 5×5 or 7×7 wiener filter) and averages the three scanned images of each film in order to reduce scanning noise. ImageJ software (National Institute of Health, Bethseda, MD) was used to extract the pixel values (PV) readings from red channel, which was used for calculation of the netOD and the dose. Our film‐based measurements of the dose, reported in this work, have a maximum uncertainty of ±1.5% in the measured PDD, following the estimation approach by Devic et al.^(47)^

### E. MOSkin measurements

A special design of MOSFET detector known as MOS*kin* for its dosimetry capabilities at skin surface and interfaces^26^, ^27^, ^52^, ^53^, ^54^, ^55^, ^56^, ^57^, ^58^ was used in this study. MOS*kins* are real‐time detectors offering water‐equivalent effective depth (WED) of measurement of 0.02 or 0.07 mm, depending on type, developed at the Centre for Medical Radiation Physics (CMRP), University of Wollongong, Australia. The MOS*kin* chip is embedded into the 0.4 mm thick KAPTON pigtail with a width of 3 mm and length about 35 cm that allow electrical connections to the small $0.6\times 0.8\times 0.35\;{\text{mm}}^{3}$ silicon chip and are all packaged in a novel design that provide a reproducible WED of measurements. Such design avoids using an epoxy bubble and makes MOS*kin* useful for placement into interfaces or confined spaces in a phantom. In this work, MOS*kin* detectors with WED of 0.02 mm were used.

The MOS*kin* was placed in a 2 mm slab of solid water, which has been grooved specifically to accommodate the detector. The MOS*kin* was carefully leveled with slab surface when installed. All the measurements were done while the MOS*kin* sensitive layer was facing the beam (“face on” configuration). To account for sensitivity variation, before and during measurement sessions, the dosimeters were periodically calibrated against the reference field.^(59)^ Each measurement point with MOS*kin* detector was repeated three times and results were averaged.

Near the interfaces, MOS*kin* PDD measurements were acquired in submillimeter depth increments using combinations of 100–400 μm thick sheets of water‐equivalent plastic. In the buildup region, MOS*kin* detector was benchmarked against Attix Parallel Plate IC (Gammex RMI) as a gold standard for the 6 and 18 MV photon fields, with field sizes ranging from $10\times 10\;{\text{cm}}^{2}$ to $40\times 40\;{\text{cm}}^{2}$ and a SSD of 100 cm demonstrating excellent agreement (within ±1.5%, results not shown here).

### F. Relative performance of different dosimetry methods and different calculations

The experimental and calculation methods, used in this study, are different and each of them has some strengths and weaknesses. Therefore, we do not claim one of the methods as the “gold standard”.

MC method simulates particle transport through the medium by randomly sampling their interaction probabilities with medium within well‐known physics principles. Thus, MC calculations are very reliable and accurate as long as used appropriately and the beam models are validated. In the literature, it has been used extensively as a dosimetric benchmark compared to alternative calculation algorithms and even against experimental measurements.

MOS*kin*, with its special packaging design, provides a very thin effective depth of measurement of 0.02 mm. It is a real‐time dosimeter and has good characteristics linearity and decent reproducibility.^53^, ^54^ MOS*kin* detector has always been used in its linear dose range by using current annealing technique^60^, ^61^ for recovery of its initial threshold values after about 30 Gy accumulated dose that warrants its linearity. However, care needs to be taken to minimize measurement uncertainties, such as voltage creep‐up effect that could introduce up to 2% error in a typical clinical dose of 2 Gy.^(62)^ Like many semiconductors, MOS*kin* exhibits temperature, energy, and angular response. The MOS*kin* temperature and creep‐up effects were minimized by taking frequent reference measurements, and by keeping the time interval between irradiation and readout small and consistent.

Gafchromic EBT2 films are near tissue‐equivalents with a very thin active layer of 0.03 mm. EBT2 film is 0.285 mm thick and has an effective depth measurements of 0.095–0.195 mm (depending on the film orientation relative to incoming beam). EBT2 was shown to have minimal energy and angular response.^39^, ^63^ However, they are not real‐time dosimeters and it could take more than a day until readings are accessible. The film dosimetry protocol contains several stages where errors and uncertainties may originate. Therefore, a well developed and consistent protocol needs to be used to minimize the errors.

Acuros XB and AAA are dose calculation algorithms optimized for fast dose calculations. Dose accuracy is, therefore, competing against short calculation time that is essential in clinical use. They share the linac head model with approximations that can impact the accuracy of dose calculations. AAA calculates the dose through convolution of photon fluence and energy deposition density function with scatter kernel that defines the lateral scattering in the phantom.^(6)^ AXB is based on solving LBTE and has been shown to produce accurate dose calculations, even in complex phantoms.^10^, ^11^

In this study, we have chosen MC calculations as a reference for the purpose of data presentation. All measurements were compared to MC, and the local differences were calculated by subtracting MC calculations from the other measurements/calculations:

(1) $$\%\Delta_{D-MC}=(Dose_{D}-Dose_{MC})$$

where *D* stands for AAA, AXB, MOS*kin*, or EBT2.

## III. RESULTS

### A. PDDs and profiles in water–air phantom

**Figure 4:**
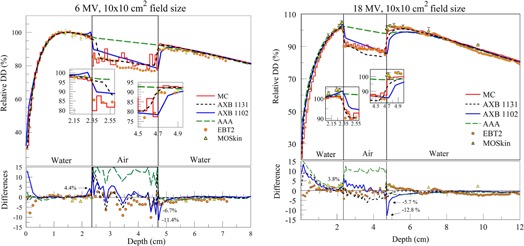
PDDs in the water–air phantom 6 MV (left) and the 18 MV (right) photon beams. Notice that inset plots have different vertical scaling.

The results for the water–air phantom are shown in Fig. 4 for 6 and 18 MV photon beams. Note that no MOS*kin* measurements were done in the buildup region, as these point‐by‐point measurements were performed only near in‐phantom interfaces. As was mentioned earlier, MOS*kin* was in excellent agreement with Attix IC for all measurements in buildup regions for 6 and 18 MV photon fields.

For 6 MV photon energy at the water–air interface AXB1102, dose calculations were in good agreement with MC, except in front of the water/air interface where a nonphysical dose buildup of 4.4% was predicted. AXB1131 removed this buildup and agreed with MC calculations within 0.7%. EBT2, MOS*kin*, and AAA were all within 2% of MC calculations. At the secondary buildup, AXB1102 underpredicted the dose by over 10%, EBT2 measurements agreed with MC within 3.6%, and all other techniques were within 2%–3% of MC. Beyond 0.2 cm from the air/water interface, the PDDs from both versions of AXB agreed with MC calculations within 1%. The average dose differences, %Δ AXB‐MC, in this region were 0.3% and 0.7% for AXB1131 and AXB1102, respectively.

For 18 MV photon energy, AXB1131, AXB1102, and AAA overpredicted MC dose in the first centimeter of the buildup region by up to 9.1%, 13.5%, and 22.3%, respectively (Fig. 4, right). Meanwhile, MOS*kin* and EBT2 measurements were in agreement with MC calculations within 3.5%–4.5% and −2.9%–2.6%, respectively. In the second buildup region, AXB1131 and AXB1102 calculations differed from Monte Carlo by up to ∼3.6% and 12.8%, respectively. However, beyond 2 mm from the distal interface, differences lowered to 1.2% and 4.4% for AXB1131 and AXB1102, respectively; MOS*kin* and EBT2 measurements agreed with MC within ∼3.0%.

**Figure 5:**
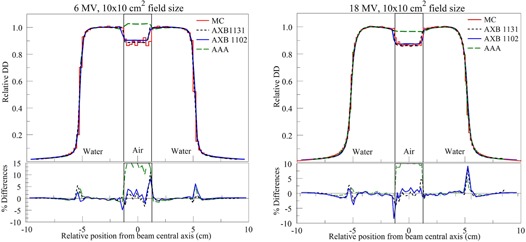
Lateral profiles through the center of the rectangular air cavity for 6 MV (left) and 18 MV (right) beams.

Figure 5 presents calculated lateral profiles taken through the center of the air gap. For both beam energies, dose calculations predicted in water by AAA, AXB1102, and AXB1131 were in good agreement with MC calculations everywhere, except penumbra and interfaces.

### B. PDDs and profiles in water–steel phantom

**Figure 6:**
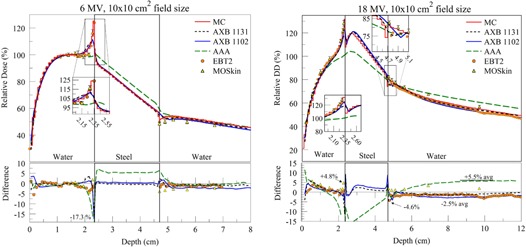
PDDs in the water–steel phantom using 6 MV photon beam (left) and the 18 MV photon beam (right). Notice that inset plots have different vertical scaling.

The relative depth doses for the water–steel phantom for 6 and 18 MV beam energies are presented in Fig. 6.

For the 6 MV beam, all calculations and measurements were in good agreement, except AAA, which failed to predict the back scatter from the high‐density heterogeneity and underestimated the dose by 25.5% compared to MC, in front of the steel/water interface. Notice in the voxel adjacent to the water/steel interface, differences of up to −15.9% and −17.3% were observed for AXB1131 and AXB1102, respectively. Meanwhile, MOS*kin* and EBT2 differed from MC calculations by up to −4.4% and −3.8%, respectively. At the distal (steel/water) interface, AXB1131, AXB1102, and AAA calculations differed to MC by up to 2.8%, 1.8%, and 6.2%, respectively. Beyond 0.2 cm from the distal interface, AXB1131 and AAA calculations agreed with MC within ∼1.5%, and AXB1102 agreed within ∼2.5%. At the same interface, MOS*kin* differed from MC by up to −3.9% directly on interface, whereas the EBT2 measurement differed from MC calculation by −1.7%.

For the 18 MV beam, in the buildup region of the water–steel phantom, agreement of measured and calculated doses was under 5% for the most part, with slightly higher differences in the first centimeter from the surface. Good agreement, within 2.0% between measurements and calculations, was found in the proximity of water/steel interface, with the exception of AAA calculations that, again, did not accurately model backscatter from steel and underestimated the dose by 28.9% in the immediate proximity of the interface. The dose calculated by AXB1131 and AXB1102 in the voxels immediately adjacent to the interface differed from MC by −11.2% and −6.8%, respectively. In the same region, EBT2 measurement was within ∼2.0% and MOS*kin* differed from MC by 4.8%. Immediately beyond to the steel/water interface, AXB1131, AXB1102, and AAA underestimated MC calculated doses by 4.6%, 4.0%, and 3.5%, respectively, and their doses in the PDD tail region differed from MC by −1.2%,−2.5%, and 5%, respectively. MOS*kin* and EBT2 measurements at the interface and in the PDD tail region were within 0.5%–3.3% and 1.5%–3.0%, respectively, compared to MC calculations.

**Figure 7:**
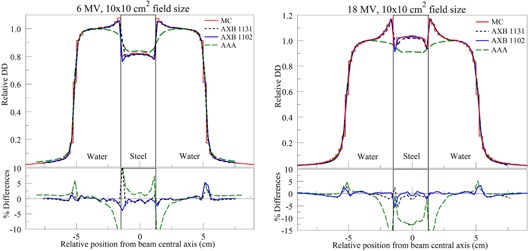
Lateral profiles through the center of the rectangular steel insert for 6 MV (left) and the 18 MV (right) beams.

Figure 7 shows the lateral profiles extracted through the center of the steel insert along the x‐axis (see Fig. 1(a)) for MC, AXB (1102 and 1131), and AAA. Lateral dose enhancement was predicted by MC and both versions of AXB near the interfaces. Both versions of AXB calculations were in agreement with MC calculations within ∼2.0%, except penumbra regions. AAA, however, underestimated the dose near the interfaces by 4.5%–12.4% and 5.5%–19.0%, respectively, for the 6 MV and 18 MV beams.

### C. PDDs in lung–water phantom

**Figure 8:**
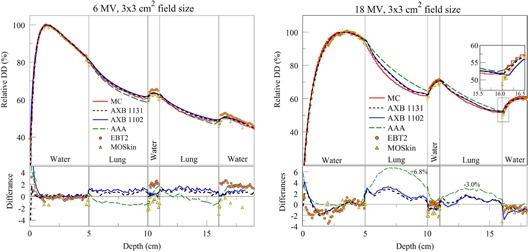
PDDs in the lung–water phantom using 6 MV (left) and the 18 MV (right) photon beams. Notice that inset plots have different vertical scaling.

PDDs for the 6 MV beam are shown in Fig. 8 (left) in the water–lung phantom. Except for the first half centimeter of the buildup region, all calculations and measurements were in agreement within 3%. All TPS algorithms were in agreement with MC within 2%. Maximum differences of 2.5% between EBT2 measurements and MC were observed in both lung–water secondary buildup regions. MOS*kin* measurements in these regions were up to 2.9% lower than MC, with the greatest differences being right at the interface. Given MC statistical uncertainty of 1% and experimental uncertainties of over 1.5% (±1.5% for EBT2 and ±2.5% for MOS*kin*), MC and measurements agreed within their combined uncertainties. Lower values of MOS*kin* directly on secondary buildup interfaces (interface 2 and 4) are partially due to much higher spatial resolution of the MOS*kin* (sensitive volume thickness is less than 1 micron and close to the interface as close as 0.02 mm) in comparison to Monte Carlo simulations (voxel size is 0.05–0.1 cm) and water‐equivalent depth of measurements 0.02 mm for used MOS*kin*. The same tendency for MOS*kin* measurements can be seen on water–lung interfaces in the builddown region, where, like on the lung–water interface, dose gradient is very steep.

PDDs for the 18 MV beam are shown in Fig. 8 (right). For the most part, MOS*kin*, EBT2 measurements, and MC calculations agreed within ∼2%. An exception was MOS*kin* measurement versus MC calculation points that were right at the lung–water interfaces. The maximum difference relative to MC was −3.1%, which is explained above. There were also few EBT2 dose points (at the depth of ~ 2 cm) where the difference exceeded 2%. However, these differences can be attributed to “noise” due to film/scanner nonuniformities that remained in the data, despite EBT2 processing as described in the Materials & Methods section.

Differences between TPS calculation algorithms and MC were larger for 18 MV compared to 6 MV. Maximum differences were observed in the buildup region, as well as lung slabs. In the upper‐lung slab, AAA, AXB1102, and AXB1131 overestimated MC dose by up to 6.8%, 3.2%, and 2.8%, respectively. In the second lung slab, both AXB versions were within 2% from MC, whereas AAA overestimated MC dose by 3%. At the second lung–water interface, agreement between all dosimetric techniques, except AXB1102, was within less than 2%. AXB1102 showed a discrepancy of −2.8% within the first half‐centimeter of the secondary buildup.

1, 2 show a summary of relative doses measured by EBT2 and MOS*kin* near interfaces. In general, differences between the two dosimeters were within ±4%, except at the water–steel interfaces in 18 MV beam, where difference of −4.6% and −7.1% were measured. These differences are attributed to the different depths of effective measurement point, combined with experimental uncertainties for the two dosimeters

**Table 1:** PDD dose‐point comparisons between EBT2 and MOS*kin* at the interfaces of water–air, water–steel, and water–lung phantoms for the 6 MV case

		*EBT2*	*MOS*kin	$\%\Delta_{EBT2-MOS\text{kin}}$
Water–air	Interface 1	97.8%	95.3%	2.5%
	Interface 2	89.9%	90.3%	−0.4%
Water–steel	Interface 1	112.7%	116.4%	−3.7%
	Interface 2	50.0%	48.4%	1.6%
Water–lung	Interface 1	84.7%	82.1%	2.6%
	Interface 2	64.3%	62.9%	1.4%
	Interface 3	65.2%	62.1%	3.1%
	Interface 4	51.8%	49.2%	2.6%

**Table 2:** PDD dose‐point comparisons between EBT2 and MOS*kin* at the interfaces of water–air, water–steel, and water–lung phantoms for the 18 MV case

		*EBT2*	*MOS*kin	$\%\Delta_{EBT2-MOS\text{kin}}$
Water–air	Interface 1	100.7%	104.5%	−3.8%
	Interface 2	99.5%	101.2%	−1.7%
Water–steel	Interface 1	123.7%	128.3%	−4.6%
	Interface 2	75.6%	82.7%	−7.1%
Water–lung	Interface 1	93.6%	94.2%	−0.6%
	Interface 2	63.5%	61.8%	1.7%
	Interface 3	65.2%	62.1%	3.1%
	Interface 4	71.7%	68.5%	3.2%

## IV. DISCUSSION

In this paper, five dosimetric techniques that include EBT2 and MOS*kin* detectors, as well as MC and Eclipse TPS (AAA and two versions of Acuros XB) calculations, have been used to measure and calculate dose profiles in three multilayer heterogeneous phantoms with water/air, water/lung, and water/steel interfaces. This combination of experimental and calculation dosimetry techniques has been used for the first time to evaluate the dose near these interfaces. The study assessed the dose from different dosimeters in nonequilibrium regions near low‐ and high‐density heterogeneities. Larger differences were found among all the dosimeters at the interfaces and the buildup regions. This is where the differences in properties of the dosimeters are highlighted by the steep dose gradients. In the following sections, results of each phantom are discussed separately.

### A. Water–air phantom

In this paper, we found that at water/air and air/water interfaces MC and AXB1131 calculations were closer to experimental measurements (EBT2 and MOS*kin*) than AAA and AXB102 for both energies. For both energies, maximum differences between all dosimetric techniques and MC calculations were observed in the secondary buildup region where AXB1102 underestimated MC calculations by 5.7% to 12.8%, while AXB1131 only underestimated it by 3.6%. Other studies^10^, ^13^, ^15^ observed similar differences at interfaces between older AXB releases and a benchmark. Bush et al.^(10)^ observed a difference up to 4.5%, just beyond 10 cm air gap, between AXB (10.0.02) and MC for a 6 MV beam. This study excluded the first voxel after the air gap where the differences were higher. Kan et al.^(13)^ reported the difference of 7.3% between AXB (10.0.28) and TLD measurement at the distal air/water interface, for a $5\times 5\;{\text{cm}}^{2}$ 6 MV beam. Stathkis^(15)^ reported differences of 3%–15% between AXB (10.0) and MC in PDD after air heterogeneity using 6 MV beam. However, all the mentioned studies used older versions than the current released version used in this study which has been confirmed to be an improvement. For the first time in this study, a comparison involves an earlier version of AXB (11.0.21) with a recent clinical release (11.0.31).

### B. Water–steel phantom

For the water–steel phantom, our results showed that all methods were in reasonable agreement as compared to MC calculations, except close to the steel insert. Lloyd and Ansbacher^(20)^ did similar work using AXB (11.0.02), but did not provide film measurement before/after the high‐density insert of the 6 MV beam. Another study by Ojala et al.^(21)^ that involved comparison of AXB (10.0.28), MC, IBA SFD, farmer IC, and EBT3. However this study did not include 18 MV beam energy, and experimental measurements were only taken after the high‐Z insert.

Our findings were consistent to previous investigations that included AAA and AXB comparison in high‐density heterogeneities, such as bone, stainless steel, and titanium alloy, in which AXB proved to be superior to AAA.^10^, ^12^, ^20^, ^21^ Our results showed that AAA differed from MC by an average of ∼5.5%, after the rectangular steel insert. This is due to inaccurate modeling of beam attenuation in the high‐density heterogeneity within the water–steel phantom. This is consistent to the Lloyd and Ansbacher study, where similar overestimation by AAA was observed after rectangular steel insert.

Our transverse dose profiles and PDDs for the water–steel phantom showed that AAA was not accurate in predicting lateral and backscatter radiation from high‐density heterogeneities. This is comparable to the findings by Lloyd and Ansbacher^(20)^ where similar underestimation of calculated dose by AAA was observed near (steel/water) interface. AXB (1131 and 1102), on the other hand, predicted the backscatter behavior, and its calculations were in a good agreement with MC and EBT2 measurement at the water/steel interfaces. However, unlike the Lloyd and Ansbacher study, our results showed differences (excluding voxels adjacent to the interfaces) of −1.6% to −5.5%, between AXB and MC at the water/steel interfaces. This might be attributed to the location of the steel insert being within the 18 MV buildup regions, at 2.35–4.7 cm depth, which makes calculations more challenging. Differences were even higher in the voxels directly adjacent to the interfaces (−6.8% to −17.3%). This could be attributed to the Eclipse built‐in intravoxel interpolation feature and phantom voxelization. Interpolated points within voxels bordering different materials provide inaccurate “interpolated” dose. Vassiliev et al.^(64)^ compared calculated dose distribution from Acuros XB and MC on a point‐to‐point basis, making sure that the matrices coincide in the spatial domain. In this study, we are interested to test AXB and AAA within the TPS package, using tools available to evaluate and compare dose profiles.

Our results showed that EBT2 and MOS*kin* measurements were in good agreement with MC calculations at the water/steel interfaces for both sets of energy. Maximum difference of ∼4.8% between experimental measurement and MC can be seen within 0.2 cm of both water/steel and steel/water interfaces. The disagreement between MC and the experimental detectors could be attributed to volume averaging due to MC scoring voxel size as compared to the smaller detection volume in MOS*kin* and EBT2.

### C. Water–lung phantom

Results for the PDD of the 6 and 18 MV beams in water–lung phantom (Fig. 8) showed that all experimental and calculation dosimetric methods, except AAA, were within ∼3% everywhere, except in the buildup and interfaces regions.

Our results, in Fig. 8, showed that AXB (1131 and 1102) were in a better agreement with MC calculation than AAA, within 2.8%–3.2% throughout the phantom for both energies. This is comparable to previous studies.^10^, ^64^ Vassiliev et al.^(64)^ reported 2.3% maximum difference between AXB and MC within lung in multilayer phantom (water‐bone‐lung‐water) using $2.5\times 2.5\times 0.35\;{\text{mm}}^{2}$ 18 MV field. Bush et al.^(10)^ found that AXB was in agreement with BEAM/DOSXYZnrc to within ±3.0% of the maximum dose within lung (0.24 $g/{\text{cm}}^{3}$) using 18 MV $4\times 4\;{\text{cm}}^{2}$ beam incident on water–lung–water phantom.

AAA calculations differed from MC by up to 6.8% within the lung for the 18 MV beam. Such large differences of AAA, compared to benchmark, were also reported in previous studies. Han et al.^(12)^ reported 17.6% as maximum relative difference between AAA and EGSnrc when using a $2.5\times 2.5\times 0.35\;{\text{mm}}^{2}$ 18 MV field in lung‐slab of a multilayer slab phantom (tissue‐bone‐lung‐tissue). Bush et al.^(10)^ reported that AAA underestimated BEAM/DOSXYZnrc by 8% within lung ($0.24\;g\;{\text{cm}}^{-1}$) using 18 MV $4\times 4\;{\text{cm}}^{2}$ beam incident on water‐lung‐water phantom. Ding et al.^(25)^ reported a 6.0% difference between MC and AAA in lung using a $3\times 3\;{\text{cm}}^{2}$, 10 MV single beam. The variations in differences between our findings and the aforementioned studies originate from the different lung density (0.3 $g/{\text{cm}}^{3}$), phantom structure, and beam configuration used in our study.

For the 6 MV, MOS*kin* measurements were up to 2.3% lower than MC at the upper water– lung interface (interface 2) and 2.9% lower at the last lung–water interface (interface 3). This is still an acceptable agreement, considering MC statistical uncertainty (∼1%) and MOS*kin* measurement uncertainty (∼2.5%). Similar agreements of MOS*kin* with EBT2 film and MC were observed for 18 MV beam, as well. This is consistent with the results reported by Ding et al.^(25)^ who used MOSFET along with MC (BEAMnrc/DOSXYZnrc) to validate AAA dose in water/lung phantom.

For both energies, EBT2 measurements were in agreement with MC calculations to within 3.0%, which support our MC model to produce accurate calculations in the tested phantoms.

## V. CONCLUSIONS

Our study showed that all dosimetric techniques, except AAA, were in good agreement (∼3%) for both photon fields used in the three phantoms for this study, except in the buildup regions and interfaces where differences were more pronounced. Also, relatively large differences (3%–6.8%) between AAA and AXB or MC in lung were observed when using higher energy (18 MV) and that is due to the differences in their dose reporting modes.

Dose differences among the dosimetric techniques were larger for the 18 MV as compared to the 6 MV photon beam. The location of the air gap and the steel insert within the buildup region of the 18 MV beam introduced extra dosimetric challenge, resulting in greater differences at 0.2–1 cm depth.

The latest version of AXB (11.0.31) showed improved agreement with MC and measurements compared to the previous version (11.0.02). Maximum differences between TP algorithms and MC were found near air/steel air/water interfaces. Differences between phantom voxelization methods used by AXB (Eclipse) and MC calculations highlighted the discrepancies near interfaces. While phantoms used in MC were produced with interfaces being between voxel boundaries, in Eclipse phantoms the interface crosses voxels and resamples densities across low‐ and high‐density materials. This reduces the accuracy of TP dose calculations at the voxel size distances from the interface.

MOS*kin* and EBT2 measurements were in good agreement with MC calculations, except at the interfaces with steep dose gradient, where differences were larger. This was due to the fact that both detectors have small detection thickness and could measure the dose very close to an interface. Dosimeter type and thickness of dosimetric sensitive volume are critical in those regions, in which very thin and tissue‐equivalent dosimeters provide more accurate dose assessment.

AAA did not predict the backscatter dose in front of the high‐density heterogeneity (steel), which resulted in a significant underestimation of the calculated dose in this region. AAA was shown to produce incorrect calculations downstream of the high‐density heterogeneity (steel), due to the inaccurate modeling of the attenuation within the steel insert.

## ACKNOWLEDGMENTS

Authors would like to thank Drs. M. Lerch, M. Petasecca, and D. Cutajar of CMRP for useful discussion and MOS*kin* probes preparation. We extend our thanks to Dr. Reid Townson for discussions on MC model configurations and for maintaining MC cluster used in MC calculations.
